# Vitreoretinal lymphoma occurring after systemic chemotherapy for primary conjunctival diffuse large B cell lymphoma: A case report

**DOI:** 10.1097/MD.0000000000027347

**Published:** 2021-10-01

**Authors:** Erina Niidome, Yoshihiko Usui, Reisuke Takahashi, Toshitaka Nagao, Hiroshi Goto

**Affiliations:** a Department of Ophthalmology, Tokyo Medical University, Tokyo, Japan; b Department of Diagnostic Pathology, Tokyo Medical University, Tokyo, Japan.

**Keywords:** conjunctival lymphoma, diffuse large B-cell lymphoma, vitreoretinal lymphoma

## Abstract

**Introduction:**

Ocular adnexal lymphoma and vitreoretinal lymphoma are rare forms of non-Hodgkin lymphoma. They are regarded as distinct disease entities due to the differences in molecular mechanism, management, and outcome. We present a rare case of conjunctival diffuse large B cell lymphoma (DLBCL) that developed to vitreoretinal lymphoma after systemic chemotherapy.

**Patient concerns:**

A 60-year-old man presented with a left salmon-colored conjunctival mass.

**Diagnosis:**

A biopsy was performed, and histopathologic examination showed DLBCL. Immunohistochemical staining was positive for CD20 with increased κ to λ light chain ratio.

**Interventions:**

Bone marrow biopsy also revealed DLBCL. Gallium-67 scintigraphy showed abnormal uptake only in the left orbital lesion. Ann Arbor stage was estimated as IV. The patient underwent systemic combination chemotherapy and immunotherapy.

**Outcomes:**

Four months after the last course of chemotherapy, primary conjunctival DLBCL relapsed, manifesting vitreous opacity. Diagnostic vitrectomy confirmed a diagnosis of vitreoretinal lymphoma.

**Lessons:**

Conjunctival DLBCL and vitreoretinal lymphoma are both DLBCL. After systemic chemotherapy for conjunctival DLBCL, the lymphoma may relapse in intraocular sites as secondary vitreoretinal lymphoma.

## 1 Introduction

Ocular adnexal lymphoma accounts for 1% to 2% of all non-Hodgkin lymphomas. The most common primary ocular adnexal lymphomas (around 75%–90%) are extranodal marginal zone B-cell lymphomas of mucosa-associated lymphatic tissue (MALT)^[1,2]^ with the appearance of salmon pink lesions, followed by rarer types of diffuse large B-cell lymphoma (DLBCL).^[3,4]^

On the other hand, vitreoretinal lymphoma (VRL) is a rare intraocular malignant disease arising in the vitreous and subretinal space, accounting for 1% of all non-Hodgkin lymphomas.^[5–7]^ The main histopathology of VRL is DLBCL.^[8,9]^ VRL is most often associated with central nervous system (CNS) lymphoma, and manifests before, after, or simultaneous with central nervous spread. More rarely, secondary vitreoretinal lymphoma may progress from systemic lymphomas in other parts of the body excluding the CNS.

Although most of the conjunctival lymphomas and intraocular lymphomas are derived from B cells, there are no reports of the occurrence of these 2 types of lymphoma in the same patient. In recent studies, secondary VRL that progresses from systemic lymphoma was found in 5% to 28% of the patients.^[10–13]^ Our search of literature found 60 patients with secondary VRL in previous reports, and the major primary lesions were in the lymph node (22 patients, 36.7%), testis (15 patients, 25%), and breast (7 patients, 11.7%).^[11–21]^

To the best of our knowledge, we report for the first time a case of VRL that occurred after chemotherapy for conjunctival DLBCL.

## 2 Case presentation

A 60-year-old man with a 1-month history of left eyelid swelling was referred by a local ophthalmologist to our department for investigation of suspected conjunctival MALT lymphoma.

**Figure 1:**
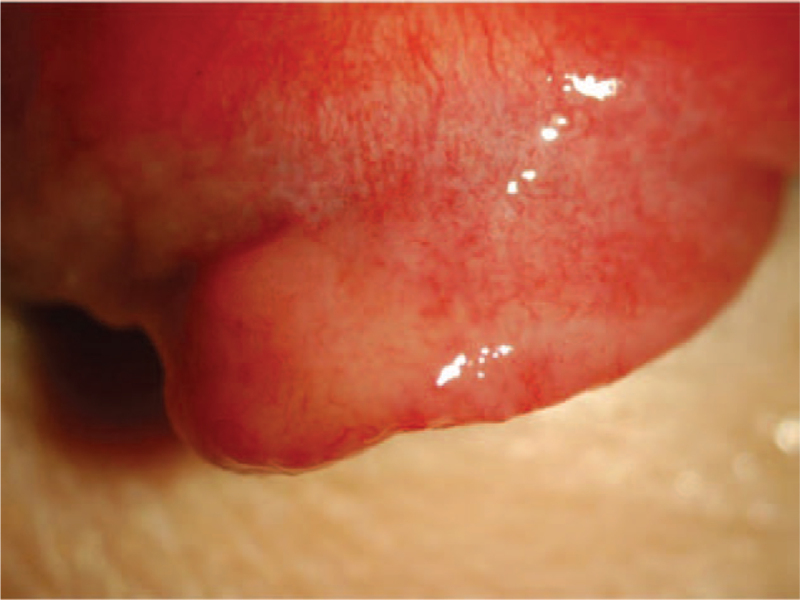
A conjunctival salmon pink lesion in the upper fornix of the left eye.

**Figure 2:**
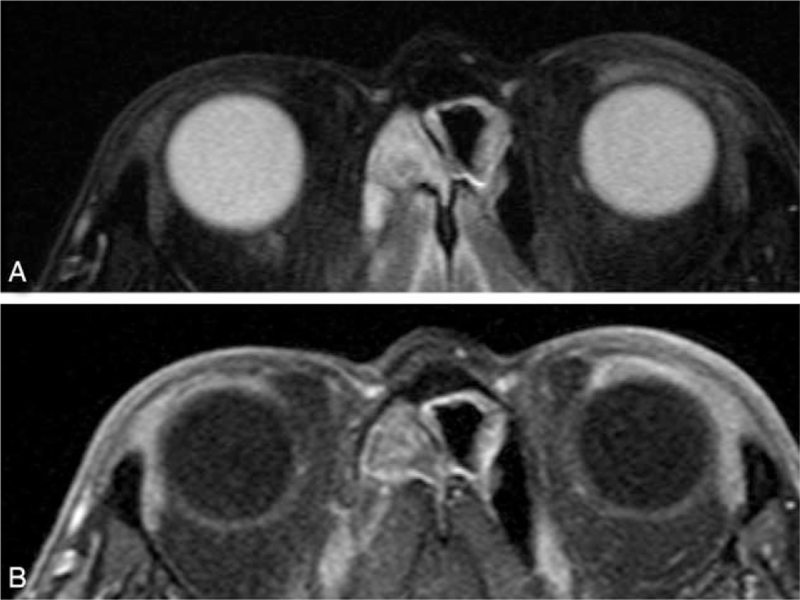
A magnetic resonance image showing the mass on the temporal side of the left eye with a contrast effect. (A) T2 weighted imaging. (B) Contrast-enhanced T1-weighted imaging.

A salmon-pink lesion was noted in the upper fornix of the left eye (Fig. 1). Magnetic resonance imaging showed a mass on the temporal side of the left orbit (Fig. 2). At presentation, his best-corrected visual acuity was 20/20 in both eyes, and the anterior segment, vitreous body, and fundus of both eyes were normal, indicating no vitreoretinal abnormalities when the patient initially presented with conjunctival lesion. Laboratory findings were as follows: white blood cell count 5 × 10^9^/L (reference range 4–11 × 10^9^/L), (neutrophils 60.0%, monocytes 4.6%, and lymphocytes 20.7%); C-reactive protein <0.30 mg/dL (reference range <0.30 mg/dL), IgG 475 mg/dL (reference range 870–1700 mg/dL), IgA 39 mg/dL (reference range 110–410 mg/dL), IgE 7.5 IU/mL (reference range 0–270 mg/dL), IgG4 19.1 mg/dL (reference range 4.5–117 mg/dL), sIL-2 receptor antibody 1890 U/mL (reference range 145–519 U/mL). Beta 2-microglobulin was 5.37 mg/L (reference range 0.64–1.56 mg/L).

**Figure 3:**
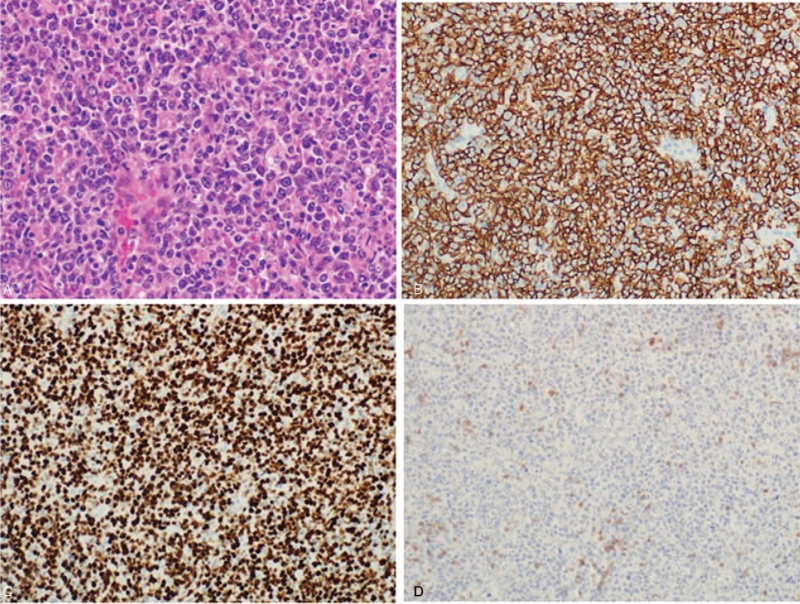
Histopathology and immunohistochemistry of the left conjunctival lesion obtained by excisional biopsy. (A) Hematoxylin-eosin staining, original magnification ×200. Immunohistochemistry studies reveal positive immunoreactivity for (B) CD20 and (C) Ki-67, and negative for (D) CD3 (original magnifications ×200).

**Figure 4:**
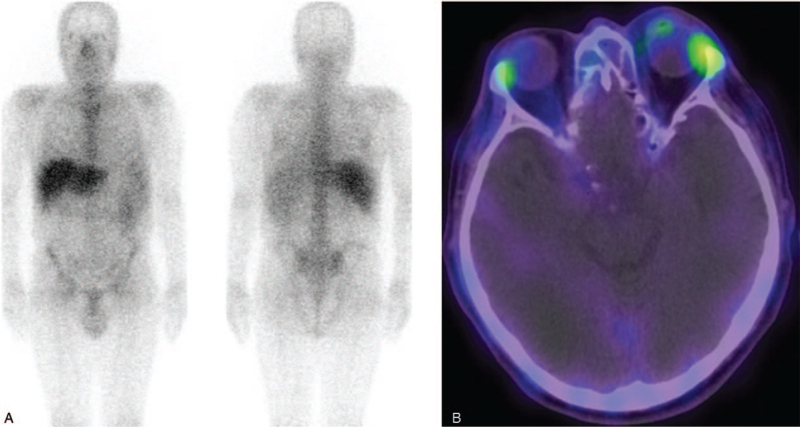
A gallium scan showed abnormal uptake only in the left orbital lesion (A, B).

An excisional biopsy through the left conjunctiva revealed DLBCL that was positive for leukocyte common antigen, CD20 antigen, and Ki-67 antigen (90%), but negative for CD3 antigen (Fig. 3A–D). Flow cytometric analysis performed on the biopsied specimen confirmed the presence of a B cell population expressing CD8, CD19, CD20, and CD22, with an increased kappa/lambda ratio (13.4). Bone marrow biopsy from the left posterior iliac crest also revealed DLBCL, and Gallium-67 scintigraphy showed abnormal uptake only in the left orbital lesion (Fig. 4). Ann Arbor stage was estimated as IV.

**Figure 5:**
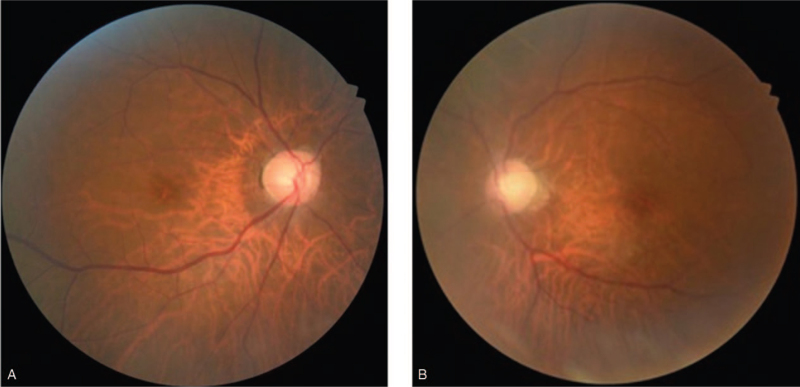
Fundus photographs 4 months after the last course of chemotherapy. Left eye shows vitritis that obscures visualization of the retina.

**Figure 6:**
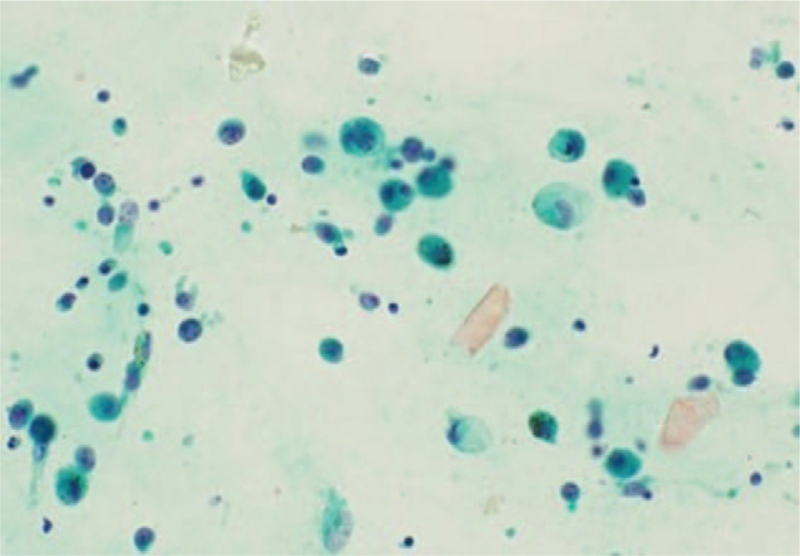
Histopathology of the vitreous biopsy sample of the left eye showing large lymphoproliferative cells (original magnifications ×400).

The patient underwent 7 courses of systemic combination chemotherapy and immunotherapy: 4 courses of rituximab, cyclophosphamide, doxorubicin hydrochloride, oncovin, and prednisolone (R-CHOP); and 3 courses of rituximab alone. He responded well to treatment; the conjunctival lesion almost disappeared after 6 months from the first visit. However, 4 months after the last course of chemotherapy, the patient complained of blurred vision in the left eye. His visual acuity was 20/20 in the right eye and 16/20 in the left eye. Slit-lamp examination of the left eye revealed grade 1+ cells with mutton-fat keratic precipitates in the anterior chamber and vitritis that obscured visualization of the retina (Fig. 5). Findings of the right eye were unremarkable. VRL was suspected from the vitreous haze finding, and a 25-gauge diagnostic pars plana vitrectomy was performed for the purpose of improving visualization of the retina and detection or exclusion of VRL. Pathological study of vitreous biopsy sample showed large lymphoproliferative cells (class V) (Fig. 6). Elevated IL-10 levels (1420 pg/mL) and increased IL-10/IL-6 ratio (35.9) were found in the vitreous sample. Flow cytometry showed a large population of CD20-positive cells with increased κ to λ light chain ratio, leading to a diagnosis of intraocular relapse of DLBCL.

After periodical intravitreal injections of methotrexate (400 μg), best-corrected visual acuity improved to 20/20 in the left eye. The therapeutic outcome further supported that the intraocular lesion was indeed the manifestation of lymphoma infiltration. No recurrence in organs of the whole body was found in 2 years, and follow-up is ongoing.

## 3 Discussion

The conjunctiva is one of the main sites involved in extranodal lymphoma, and the major histopathologic type is MALT lymphoma.^[1–3,22–25]^ Extranodal marginal zone lymphoma accounts for 68% to 80% of conjunctival lymphomas, whereas DLBCL accounts for only approximately 3% to 5%.^[23,24]^ Shields et al^[25]^ reported that lymph nodes, abdomen, and bone marrow are the most common lesions of systemic involvements of conjunctival lymphomas including all histopathological subtypes. In contrast to vitreoretinal lymphoma, primary conjunctival lymphoma rarely develops CNS involvement. Low-dose external beam radiation therapy is the standard treatment for low-grade lymphoma that is isolated to the conjunctiva,^[26,27]^ whereas systemic treatment such as rituximab plus cyclophosphamide, doxorubicin, vincristine, and prednisone (R-CHOP) is needed in cases of aggressive histopathological subtypes including DLBCL, bilateral disease, or conjunctival lymphoma accompanied by active systemic involvement.^[27]^ The recurrence rate of conjunctival DLBCL was reported as high as around 67%, and the average time to recurrence was as short as 10 months (range 2–23 months).^[22]^ Kirkegaard et al^[22]^ reported that almost all recurrent cases of conjunctival DLBCL had extraorbital lesions, whereas most of the recurrent cases of conjunctival extranodal marginal zone lymphoma were localized in the ocular appendage.

Relapse of the primary conjunctival DLBCL in our patient manifested as vitreous opacity, which is the hallmark of VRL. Even after 7 courses of combination systemic chemotherapy, the onset of VRL was not prevented in this patient. Therefore, this case highlights the possibility that lymphoma cells may have escaped the effect of systemic chemotherapy and survived in the intraocular space that is an immune-privileged site and isolated from the rest of the body by the blood-ocular barrier.

VRL is classified as primary when lymphoma originates in the eye and the CNS, and as secondary when lymphoma originating in organs other than the CNS spreads to the eye. Kimura et al^[28]^ reported that VRL with CNS involvement was most common, found in 60.8% of the patients. Also, Mochizuki et al reported that 15% to 25% of patients with primary CNS lymphoma (PCNSL) had ocular involvement at the time of diagnosis of PCNSL, and 25% of patients with PCNSL without ocular involvement at the time of diagnosis developed VRL later.^[5]^ Most cases of secondary VRL develop from extraocular primary DLBCL lesions. VRL secondary to systemic lymphoma was found in 5% to 29% of patients with VRL.^[10–13]^ We found 60 patients with secondary VRL in a literature search on PubMed, and the major primary lesions were located in the lymph node (22 patients, 36.7%), testis (15 patients, 25%), and breast (7 patients, 11.7%).^[11–21]^ However, there are no reports of secondary VRL occurring after chemotherapy for conjunctival DLBCL. In general, conjunctival lymphoma has few recurrences and is often followed clinically with a long interval. This case indicates the need to examine and treat conjunctival DLBCL considering the possibility of recurrence as intraocular lesions. Ophthalmologists should note that conjunctival DLBCL may develop and relapse in the eye after systemic chemotherapy.

## Author contributions

EN and YU were major contributors to the drafting of the manuscript. RT and TN interpreted the pathological data. HG reviewed and edited the manuscript. All authors reviewed and approved the final manuscript.

**Conceptualization:** Yoshihiko Usui.

**Data curation:** Erina Niidome, Yoshihiko Usui, Reisuke Takahashi.

**Formal analysis:** Yoshihiko Usui.

**Funding acquisition:** Yoshihiko Usui.

**Investigation:** Erina Niidome, Yoshihiko Usui.

**Methodology:** Yoshihiko Usui.

**Project administration:** Yoshihiko Usui.

**Resources:** Yoshihiko Usui.

**Software:** Yoshihiko Usui.

**Supervision:** Yoshihiko Usui, Toshitaka Nagao, Hiroshi Goto.

**Validation:** Yoshihiko Usui.

**Visualization:** Erina Niidome, Yoshihiko Usui, Reisuke Takahashi.

**Writing – original draft:** Erina Niidome, Yoshihiko Usui.

**Writing – review & editing:** Yoshihiko Usui, Hiroshi Goto.
